# Engineering of Self-Assembled Fibronectin Matrix Protein and Its Effects on Mesenchymal Stem Cells

**DOI:** 10.3390/ijms160819645

**Published:** 2015-08-19

**Authors:** Ye-Rang Yun, Le B. Hang Pham, Yie-Ri Yoo, Sujin Lee, Hae-Won Kim, Jun-Hyeog Jang

**Affiliations:** 1 Department of Nanobiomedical Science and BK21 PLUS NBM Global Research Center for Regenerative Medicine, Dankook University, Cheonan 330-714, Korea; E-Mail: yunyerang@dankook.ac.kr; 2 Institute of Tissue Regeneration Engineering (ITREN), Dankook University, Cheonan 330-714, Korea; 3 Department of Biochemistry, Inha University School of Medicine, Incheon 400-712, Korea; E-Mails: plbhang@gmail.com (L.B.H.P.); yieri0214@naver.com (Y.-R.Y.); sujin2manse@naver.com (S.L.); 4 Department of Biomaterials Science, College of Dentistry, Dankook University, Cheonan 330-714, Korea

**Keywords:** fibronectin, peptide amphiphile, rat mesenchymal stem cells, cellular activity

## Abstract

Fibronectin (FN) contributes to cell adhesion, proliferation, and differentiation in various cell types. To enhance the activity of fibronectin at the sites of focal adhesion, we engineered a novel recombinant fibronectin (FNIII10) fragment connected to the peptide amphiphile sequence (PA), LLLLLLCCCGGDS. In this study, the effects of FNIII10-PA on rat mesenchymal stem cells (rMSCs) were compared with those of FNIII10. FNIII10-PA showed the prominent protein adhesion activity. In addition, FNIII10-PA showed a significantly higher effect on adhesion, proliferation, and differentiation of rMSCs than FNIII10. Taken together, the FNIII10-containing self-assembled sequence enhanced rMSCs adhesion, proliferation, and differentiation.

## 1. Introduction

Recently, the interest in self-assembly is increasing in various tissue engineering areas. Self-assembly is defined as the organized structure or pattern of many components, such as phospholipids, peptides, or proteins [1,2]. Of the possible self-assembled components, self-assembled peptides serve as building blocks due to their small size, ease of synthesis, and physical and chemical stability [3]. In addition, self-assembled peptides exhibit biocompatibility and molecular recognition [4]. Designed self-assembled peptides form fibril, nanotube, and nanosphere structures under solvent, temperature, and pH condition [5,6]. Based on these characteristics, self-assembled peptides are widely studied in tissue engineering, as well material science [7]. For instance, octapeptide, FEFEFKFK (F, phenylalanine; E, glutamic acid; K, lysine) formed self-supporting hydrogels and supported viability, morphology retention, and collagen type II deposition of bovine chondrocytes [8]. In other study, Gelain *et al.* designed RADA16 (Ac–RADARADARADARADA–COHN_2_), RADA16-bone marrow homing peptide 1 (BMHP1), RADA16-bone marrow homing peptide 2 (BMHP2), and controlled the three-demensional culture of neural stem cells [9]. Arg–Gly–Asp (RGD) peptide has also been extensively studied. The application of RGD peptide to polylactide (PLA) or poly (ethylene oxide) (PEO) enhanced cell functions, such as adhesion and migration [10,11]. In addition, small molecules, such as ECM, regulate the surface of biomaterials, leading to influence on stem cell culture and differentiation [12]. Thus, self-assembled peptides have different functions according to the sequence design.

Interestingly, fibronectin (FN) has self-assembled characteristics. This is attributed to it having RGD in the FN structure [13]. FN is a major component of the extracellular matrix (ECM) and its structures are classified as type I, II or III [14,15]. FN type III has specific domains, such as a central cell binding domain (CCBD) and a heparin binding domain (HBD). In particular, CCBD containing RGD is an essential domain that confers cell adhesion [16]. FN commonly promotes cell proliferation, migration, and differentiation, as well as cell adhesion [15,17,18]. We previously reported that various recombinant FN type III domain fragments showed cell adhesion activity. Furthermore, we showed that this cell adhesive activity is dependent on FN type III domain fragments. FNIII9-10 commonly promoted adhesion, proliferation, and the differentiation of osteoblasts [19,20]. In a previous study, FNIII12-14 showed lower cell adhesive activity than FNIII8-10 [21]. Somewhat surprisingly, FNIII8-14 demonstrated the highest cell adhesive activity [18].

Here, we designed the amphiphile sequence and constructed FNIII10-PA. In the present study, we investigated the effect of FNIII10-PA on rMSCs compared to FNIII10. The protein adhesion activity and the effects of FNIII10-PA on the rMSCs adhesion and proliferation were investigated. Furthermore, the osteogenic differentiation activities of FNIII10-PA on rMSCs were investigated by real-time PCR.

## 2. Results and Discussion

### 2.1. Expression and Purification of FNIII10-PA in Escherichia coli (E. coli)

In this study, we constructed and purified FNIII10-PA in *E. coli* and used purified FNIII10 as a control. FNIII10 and FNIII10-PA were purified by using a nickel–nitrilotriacetic acid resin column. The yields for purified FNIII10 and FNIII10-PA from 1 liter culture were 1.2 and 1.3 mg/mL, respectively. Final purity was approximately 98%.

**Figure 1:**
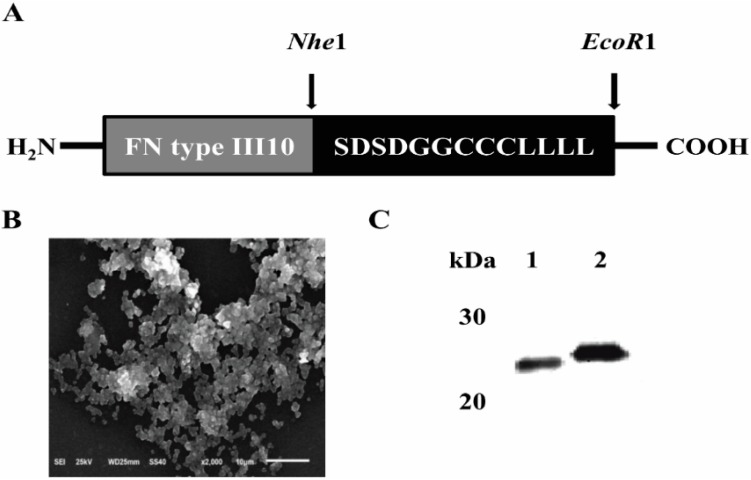
Structure (**A**), SEM image (**B**) and molecular weight (**C**) of FNIII10-PA. Peptide amphiphile sequence (PA), LLLLLLCCCGGDS (L, leucine; C, cysteine; G, glycine; D, aspartic acid; S, serine) was connected with fibronectin type III10. The molecular weight of FNIII10 and FNIII10-PA are approximately 25 (Lane 1) and 27 kDa (Lane 2).

Figure 1B showed the morphology of FNIII10-PA. FNIII10-PA was self-assembled as a fibrous structure and condensed.

The molecular weights of FNIII10 and FNIII10-PA were determined by Western blotting. As shown in Figure 1C, the molecular weights of FNIII10 and FNIII10-PA were approximately 25 and 27 kDa, respectively. As was expected, the difference of molecular weight was due to the designed amphiphile sequence.

### 2.2. Protein Adhesion Activity of FNIII10-PA

**Figure 2:**
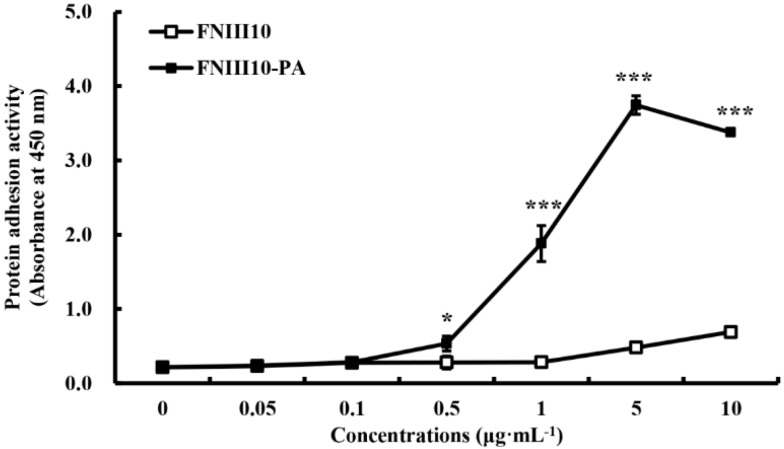
Protein adhesion activity of FNIII10-PA. Twenty-four well plates were coated with various concentrations of FNIII10 or FNIII10-PA overnight at 4 °C. After the addition of antibody, Turbo-TMB ELISA was added. Protein adhesion activities are expressed as mean ± SD (*n* = 3). * *p* < 0.05 and *** *p* < 0.001.

Figure 2 showed the protein adhesion activities of FNIII10 and FNIII10-PA. The protein adhesion activity of FNIII10-PA was dose-dependently increased. At over 0.5 μg·mL^−1^, the protein adhesion activity of FNIII10-PA was significantly higher than that of FNIII10 (* *p* < 0.05 and *** *p* < 0.001). In particular, FNIII10-PA at 5 μg·mL^−1^ dramatically increased protein adhesion (*** *p* < 0.001). FN is known to be a pivotal adhesive protein and to promote cell adhesion and, therefore, FN is widely used with materials to improve cell adhesion, as well as protein adhesion. For example, FN on poly (vinyl alcohol) hydrogels has been reported to enhance fibroblast proliferation, migration, and adhesion [22], and FN on functionalized hydroxyapatite was found to improve dermal fibroblast attachment [23]. FNIII10 containing PA promoted protein adhesive activity of FNIII10.

### 2.3. rMSCs Adhesion Activity Promotion by FNIII10-PA

**Figure 3:**
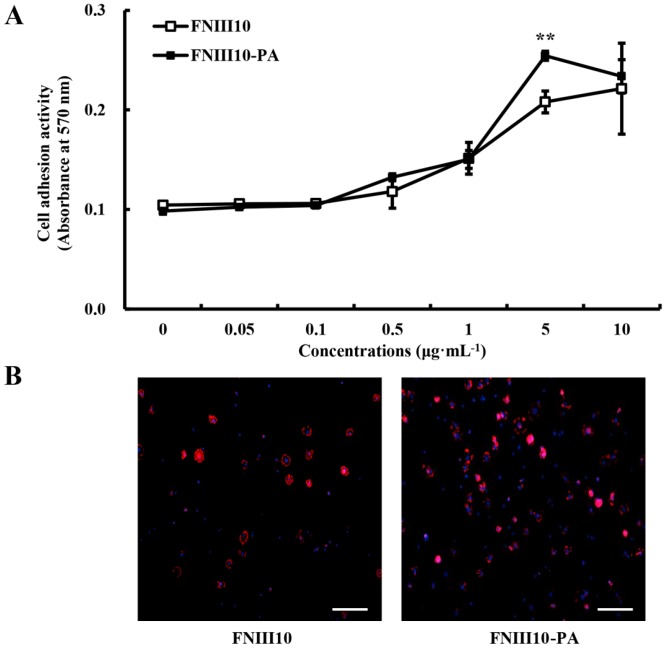
Cell adhesion activities of FNIII10-PA on rMSCs. Twenty-four well plates were coated with various concentrations of FNIII10 or FNIII10-PA overnight at 4 °C. Cells were seeded at a density of 1 × 10^5^ cells/well on plates and incubated for 1 h at 37 °C. (**A**) Cell adhesion activities were measured by crystal violate assay and expressed as mean ± SD (*n* = 3). ** *p* < 0.005; (**B**) CLSM images (nuclei in blue and F-actin in red). Scale bar, 50 μm.

To examine the effect of FNIII10-PA on cell adhesion, rMSCs were cultured on 24-well plates coated with FNIII10 or FNIII10-PA in FBS-free medium for 1 h. FNIII10-PA showed dose-dependent cell adhesion activity, as shown Figure 3A. The cell adhesion activity of FNIII10-PA at 5 μg·mL^−1^ was significantly greater than that of FNIII10 (** *p* < 0.005; Figure 3A). As shown in Figure 3B, FNIII10-PA at 5 μg·mL^−1^ increased rMSC adhesion compared to the FNIII10 through DAPI and F-actin staining. Many studies have demonstrated the cell adhesion activity of FN itself [24,25,26]. Based on protein adhesion and cell adhesion data, FNIII10-PA at μg·mL^−1^ was used in subsequent experiments.

### 2.4. The Promotion of rMSCs Proliferation Activity by FNIII10-PA

**Figure 4:**
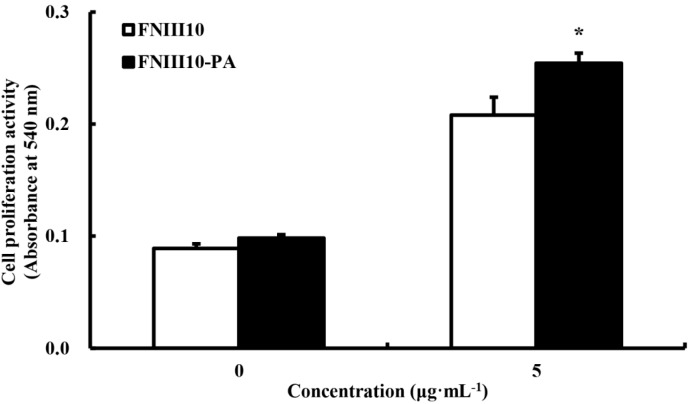
Cell proliferation activities of FNIII10-PA on rMSCs at 5 days. Twenty-four well plates were coated with 5 μg·mL^−1^ of FNIII10 or FNIII10-PA overnight at 4 °C. Cells were seeded at a density of 1 × 10^5^ cells/well on the plates and incubated for 5 days at 37 °C. Formazan absorbance was used as a measure of cell proliferation. Cell proliferation activities are presented as means ± SD (*n* = 3). * *p* < 0.05.

Cell proliferation was examined using a MTT assay. At day 5, the cell proliferative activity of FNIII10-PA was significantly greater than FNIII10 (* *p* < 0.05; Figure 4). These results are entirely consistent with our earlier studies. FN promoted adhesion, viability, and proliferation of MSC to electrospun polyethylene terephthalate (PET) and polyurethane (PU) [27]. Therefore, research of FNIII10-PA potentiates to improve with materials in tissue engineering.

### 2.5. Osteogenic Differentiation Gene Eexpression by FNIII10-PA

**Figure 5:**
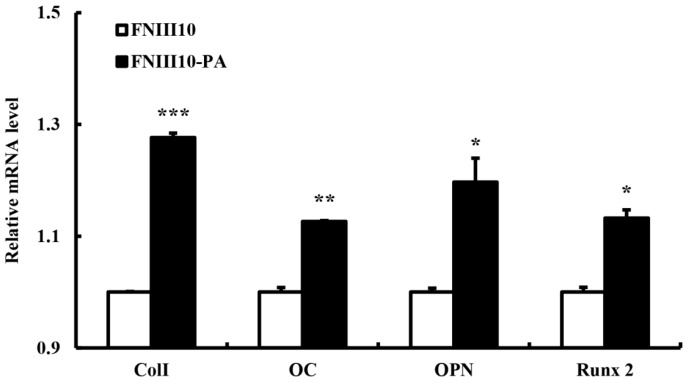
Osteogenic differentiation activities of FNIII10-PA on rMSCs at 7 days. Twenty-four well plates were coated with 5 μg·mL^−1^ of FNIII10 or FNIII10-PA overnight at 4 °C. Cells were seeded at a density of 1 × 10^5^ cells/well on plates and incubated for 7 days at 37 °C. On day 7, quantitative real-time PCR results were analyzed. * *p* < 0.05, ** *p* < 0.005, and *** *p* < 0.001.

To examine the osteogenic differentiation activity of FNIII10-PA, the mRNA levels of osteogenic differentiation gene markers, that is, *Col I*, *OC*, *OPN*, and *Runx2*, were measured by real-time PCR. Among the five most common types, *Col I* is found in skin, tendons, vascular ligature, organs, and bones. Particularly, *Col I* is a main component of the organic part of bone. Furthermore, a deficiency of Col I cause osteogenesis imperfecta [28]. *OC* is secreted by osteoblasts and participates in bone mineralization. *OC* is used as a marker of bone formation [29], as is *OPN*, whereas *Runx2* is a crucial transcription factor involved in osteogenic differentiation [30,31]. In the present study, FNIII10-PA induced the osteogenic differentiation by up-regulation of all genes. The gene expressions of *Col I*, *OC* and *Runx2* were significantly up-regulated by FNIII10-PA at 7 days (* *p* < 0.05, ** *p* < 0.005, and *** *p* < 0.001; Figure 5). In particular, FNIII10-PA significantly increased *Col I* expression (*** *p* < 0.001). In consistent with our results, Zhang *et al.* reported that FN exhibits the osteogenic differentiation activity [32,33]. Our qPCR results showed that FNIII10-PA promoted the osteogenic differentiation of rMSCs moreso than FNIII10 itself.

## 3. Experimental Section

### 3.1. Construction of Expression Plasmids

Amphipathic peptide has hydrophobic and hydrophilic regions in its structure. Thus, amphipathic peptides strongly tend to self-assemble one-dimensionally, leading to be amyloid-like fibril structures [34]. In consideration of this characteristic, we designed a peptide amphiphile sequence consisting of a hydrophobic and hydrophilic peptide with FN type III10 (FNIII10-PA). As shown in Figure 1A, the LLLLLLCCCGGDS (L, leucine; C, cysteine; G, glycine; D, aspartic acid; S, serine) was designed. To construct FNIII10-PA, the PA sequence was amplified using the following primers, 5′-ATTGATTATTTGCACGGCGT-3′, and 5′-TTAGAATTCTTAAAACAGCAGCAGCAGCAGAAACAGCAGGCAGCAGCAGCCGCCATCGCTATCGCTGCCGGTACCGCCCTCGAG-3′. PCR was performed over 30 amplification cycles (1 min at 55 °C annealing, 1 min at 72 °C extension, and 1 min at 94 °C denaturation). Amplified PCR products were digested with *NheI* and *EcoRI*, and after digestion, PCR products were ligated into pBAD-HisB-FNIII10, pBAD-HisB-FNIII10-PA to give the final constructs.

### 3.2. Protein Expression and Purification

After transformation into TOP10 *Escherichia coli* (*E. coli*), cells were grown in Luria-bertani (LB) medium containing ampicillin overnight at 37 °C. When the absorbance of cultures reached 0.6 (*A*_600_), induction was initiated with 0.1% (*w*/*v*) l-arabinose, and cells were incubated at 20 °C for 6 h. Bacteria were pelleted by centrifugation at 6000× *g* for 10 min, lysed, and sonicated. A soluble extract was prepared by centrifugation for 30 min at 14,000× *g* in a refrigerated centrifuge, and the supernatant so obtained was purified using a nickel-nitrilotriacetic acid resin (Invitrogen, Carlsbad, CA, USA).

The purities of FNIII10 and FNIII10-PA were examined under denaturing conditions by Coomassie Brilliant Blue staining on a 12% (*v*/*v*) sodium dodecyl sulfate-polyacrylamide gel (SDS-PAGE). Western blotting was performed using a peroxidase conjugate of a monoclonal anti-polyhistidine antibody (sc-8036 HRP, Santa Cruz Biotechnology, Santa Cruz, CA, USA) to confirm the expression of the recombinant fusion protein. Molecular sizes were confirmed by comparison *versus* the migration of pre-stained protein marker (Elpis Biotech, Daejeon, Korea) run in parallel lanes.

### 3.3. FNIII10-PA Morphology

After purification of FNIII10-PA, we observed the structure of FNIII10-PA under the condition of pH 5 via scanning electron microscopy (SEM) at an accelerating voltage of 15 kV after fixing with glutaraldehyde (2.5%), dehydrating with a graded series of ethanol (75%, 90%, 95%, and 100% (*v*/*v*) for 10 min each), and treating with hexamethyldisilazane and coating with platinum.

### 3.4. Protein Adhesion Assay

To evaluate protein adhesion activity, 24-well plates were coated only once with FNIII10 or FNIII10-PA (0–10 μg·mL^−1^) overnight at 4 °C. After incubation, each well was washed with phosphate-buffered saline (PBS). Wells were then blocked with 1% (*w*/*v*) BSA solution for 1 h at room temperature (RT). After washing with PBS, a peroxidase conjugate of a monoclonal anti-polyhistidine antibody was added and incubated for 1 h at RT. After washing with TBS-T, 200 μL Turbo TMB-ELISA was added and incubated for 30 min at RT. H_2_SO_4_ (100 μL, 2 M) was then added to stop the reaction. The absorbance of each well was read at 450 nm using a microplate reader. Protein adhesion assay was performed in negative control group (un-treated), positive control group (FNIII10), and experimental group (FNIII10-PA). The value of negative control was approximately equal to the value of FNIII10 and FNIII10-PA. Hence, results were expressed as the comparison between FNIII10 and FNIII10-PA without negative control.

### 3.5. Cell Isolation and Culture

To isolate rat mesenchymal stem cells (rMSCs), male adult Sprague-Dawley rats (4–5 weeks old, 180–200 g) were purchased. rMSCs were isolated from bone marrow of rat femurs and tibias, according to the code of practice for the care and use of animals for scientific purposes approved by Animal Ethics Committee, Dankook University. The bone marrow was flushed with α modified Eagle’s medium (a-MEM; Gibco, Carlsbad, CA, USA) supplemented with 10% heat-inactivated fetal bovine serum (FBS; Gibco, Carlsbad, CA, USA), and 1% penicillin–streptomycin using 10 mL syringes and filtered with a 40-μm nylon filter (Cell strainer, BD Biosciences, Spark, MD, USA). rMSCs were cultured in α-MEM containing 10% FBS, 100 units/mL penicillin G sodium, 100 mg/mL streptomycin sulfate, and 0.25 mg·mL^−1^ amphotericin B (Invitrogen, Carlsbad, CA, USA) in a 5% CO_2_ atmosphere at 37 °C. Confluent cells were detached with 0.25% trypsin-EDTA for 5 min, and aliquots were subcultured. rMSCs maintained for three passages were used for further cell adhesion, proliferation, and differentiation studies.

### 3.6. Cell Adhesion Assay

Cell adhesion activity was measured using the crystal violet assay. Twenty-four well plates were coated only once with FNIII10 or FNIII10-PA (0–10 μg·mL^−1^) overnight at 4 °C. After incubation, each well was washed with Dulbecco’s phosphate-buffered saline (DPBS), and then blocked with 1% (*w*/*v*) bovine serum albumin (BSA) solution for 30 min. rMSCs, prepared in α-MEM serum-free medium at 1 × 10^5^ cells/well, were seeded in each plate and, 30 min later, adherent cells were washed twice with DPBS and fixed with 3.7% (*w*/*v*) formalin solution for 15 min at room temperature. Cells were then stained with 0.25% (*w*/*v*) crystal violet (Sigma, St. Louis, MO, USA) in 2% (*v*/*v*) ethanol/water for 1 h at 37 °C, and gently washed 3 times with DPBS. Cells were then lysed with 2% SDS solution and transferred to 96-well plates. Absorbances were read at 570 nm using a microplate reader. Results were expressed as the comparison between FNIII10 and FNIII10-PA.

### 3.7. DAPI and F-Actin Staining

To observe the adhesion effect of rMSCs by FNIII10-PA, F-actin and DAPI staining were performed. Cells were seeded with 1 × 10^5^ cells/well. After incubation for 1 h, cells were fixed with 4% paraformaldehyde, the cells were permeabilized with 0.2% Triton-X100. Cells were blocked with 5% BSA solution for 1 h after washing. Then, cells were stained with phalloidin (A22283, Invitrogen, Carlsbad, CA, USA) to stain F-actin and the nuclei were stained with DAPI (P36935, Invitrogen, Carlsbad, CA, USA). The fluorescence image was obtained by confocal laser scanning microscopy (CLSM, Carl Zeiss 510L, Oberkochen, Germany).

### 3.8. Cell Proliferation Assay

Cell-proliferating activity was assessed using a (3-(4,5-dimethylthiazol-2-yl)-2,5-diphenyltetrazolium bromide) MTT viability assay, which measures the metabolic activity of viable cells, and was used for the indirect cell quantification according to the manufacturer’s instructions (Promega, Madison, WI, USA). FNIII10 and FNIII10-PA protein solutions were coated at 5 μg·mL^−1^ on 24-well plates, and then rMSCs were plated at a density of 1 × 10^5^ cells/well and incubated for 5 days at 37 °C. Cells were then washed three times with DPBS and 500 μL of MTT (5 mg·mL^−1^ in PBS) was added to each well. After 4 h of incubation, media were removed and formazan crystals were dissolved in 200 μL dimethyl sulfoxide (DMSO). Well absorbances were read at 540 nm using a microplate reader. Cell proliferation assay was performed in negative control group (un-treated), positive control group (FNIII10), and experimental group (FNIII10-PA). After identifying the effect of FNIII10 and FNIII10-PA as compared with negative control, results were expressed as the comparison between FNIII10 and FNIII10-PA.

### 3.9. Quantitative Real-Time PCR Analysis

FNIII10 or FNIII10-PA protein was coated at 5 μg·mL^−1^ onto 24-well plates, and then, rMSCs were plated at a density of 1 × 10^5^ cells/well and incubated for 7 days at 37 °C. Total RNA was extracted using an Easy-spin RNA Extraction kit (iNtRON, Seoul, Korea), and the cDNA was synthesized. The overexpressions of genes such as collagen type I (*Col I*), osteocalcin (*OC*), osteopontin (*OPN*), and runt-related transcription factor 2 (*Runx2*) were confirmed by quantitative real-time PCR.

All real-time PCR analyses were performed using an ABI Step One real-time PCR system. Each reaction was performed in a 20 μL reaction mixture containing 0.1 µm of each primer, 10 µL of 2× SYBR Green PCR master mix (Applied Biosystems, including AmpliTaq Gold DNA polymerase in buffer, a dNTP mix, SYBR Green I dye, Rox dye, and 10 mM MgCl_2_), and 1 µL of template cDNA. The C_t_ (cycle threshold) value for each gene was determined using the automated threshold analysis function in the ABI instrument and normalized with respect to C_t__(GAPDH)_ to obtain dC_t_ (d*C*_t_ = C_t(GAPDH)_ − C_t(specific gene)_). Finally, *C*_t_ value of FNIII10-PA was normalized by the *C*_t_ value of FNIII10. The primers used for quantitative PCR are shown in Table 1. The qPCR results were also expressed as the comparison between FNIII10 and FNIII10-PA.

**Table 1:** Sequences of primers used for real-time PCR.

Genes	Forward Primer	Reverse Primer
*GAPDH*	5′-TGGAAGGACTCATGACCACA-3′	5′-TTCAGCTCAGGGATGACCTT-3′
*Col I*	5′-CTGGCAAGAACGGAGATGAT-3′	5′-TTAGGACCAGCAGGACCAGT-3′
*OC*	5′-CATCACTGCCACCCAGAAGAC-3′	5′-CAGTGGATGCAGGGATGATGT-3′
*OPN*	5′-CCAATGAAAGCCATGACCAC-3′	5′-CGACTGTAGGGACGATTGGA-3′
*Runx2*	5′-CGCCCCTCCCTGAACTCT-3′	5′-TGCCTGCCTGGGATCTGTA-3′

### 3.10. Statistical Analysis

Experiments were conducted in triplicate. Experimental results were expressed as mean ± standard deviation (SD). The Student’s *t*-test with paired data sets was used to determine the significances of differences between two groups. Statistical significance was accepted for *p* values <0.05.

## 4. Conclusions

In summary, we found that FNIII10-PA containing the amphiphile sequence dramatically increased protein adhesion activity. Cell adhesion and proliferation of rMSCs were also enhanced by FNIII10-PA compared to FNIII10. Interestingly, FNIII10-PA induced the osteogenic differentiation of rMSCs in all genes. Consequently, the amphiphile sequence of FNIII10-PA enhances the proliferation, differentiation, and adhesion of rMSCs. Further, FNIII10-PA potentiates utilization for tissue engineering with various materials, as well as FNIII10-PA alone.
